# Post Synthetic Modification of Planar Antiaromatic Hexaphyrin (1.0.1.0.1.0) by Regio-Selective, Sequential S_N_Ar

**DOI:** 10.3390/molecules26041025

**Published:** 2021-02-15

**Authors:** Ranjan Dutta, Brijesh Chandra, Seong-Jin Hong, Yeonju Park, Young Mee Jung, Chang-Hee Lee

**Affiliations:** 1 Department of Chemistry, Kangwon National University, Chuncheon 24341, Korea; ranjand.chem@gmail.com (R.D.); brijesh.chem@gmail.com (B.C.); sjhong120@kangwon.ac.kr (S.-J.H.); ymjung@kangwon.ac.kr (Y.M.J.); 2 Kangwon Radiation Convergence Research Support Center, Kangwon National University, Chuncheon 24341, Korea; yeonju4453@kangwon.ac.kr

**Keywords:** hexaphyrin (1.0.1.0.1.0), antiaromatic, 24π-electron system, nucleophilic aromatic substitution, aromatic, benzodioxane

## Abstract

In spite of unique structural, spectroscopic and redox properties, the synthetic variants of the planar, antiaromatic hexaphyrin (1.0.1.0.1.0) derivatives **2**, has been limited due to the low yields and difficulty in access to the starting material. A chemical modification of the *meso*-substituents could be good alternative overcoming the synthetic barrier. Herein, we report a regio-selective nucleophilic aromatic substitution (S_N_Ar) of *meso*-pentafluorophenyl group in rosarrin **2** with catechol. The reaction afforded benzodioxane fused rosarrin **3** as single product with high yield. The intrinsic antiaromatic character of the starting rosarrin **2** retained throughout the reactions. Clean, two electron reduction was achieved by treatment of **3** with SnCl_2_•2H_2_O affording 26π-electron aromatic rosarrin **4**. The synthesized compounds exhibited noticeable changes in photophysical and redox properties compared with starting rosarrin **2**.

## 1. Introduction

**Figure 1:**
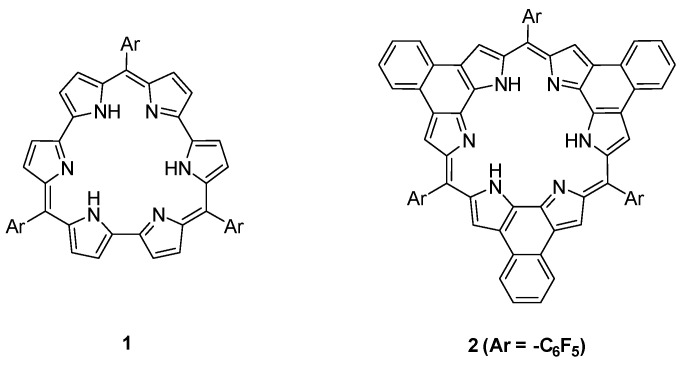
Chemical structures of rosarrin **1**, **2**.

The Expanded porphyrinoids specially hexaphyrins, have been synthesized and being explored extensively owing to its flexible structure, coordination ability and unique photophysical properties [1,2,3,4,5]. The flexible, non-planar structural property affects the overall aromaticity of the hexapyrrolic macrocycles as well as photophysical properties. These hexapyrrolic macrocycles have been modified by many ways and has been explored in terms of coordination ability, changes in redox behavior, molecular orbital perturbations. Efforts are still on going in these directions. While, the conformationally locked hexaphyrin, developed by Osuka group has exhibited rigid rectangular conformation. The compounds displayed strong Hückel’s aromaticity or antiaromaticity depending on the number of π-electrons [6]. The hexaphyrin[1.0.1.0.1.0] or rosarrin (**1**) (Figure 1) is an unique class of expanded porphyrin and it adopts non-planar geometry with weaker antiaromatic character [7]. However, the β,β′-annulated rosarrin (**2**) was a planar, fully conjugated, stable antiaromatic 24π-electron system. The rosarrin **2**, bearing pentafluorophenyl group at the *meso*-position, displayed reversible redox state changes from antiaromatic 24π electron system to aromatic 26π electron system upon protonation. The redox switching was proceeded via proton coupled electron transfer (PCET) mechanism [8]. Metallation of rosarrin **2** with Rh(I) has led to the coordination of two Rh(I) on opposite side of the macrocyclic core with Rh(I)-Rh(I) interaction [9].

Successful modifications at periphery and *meso*-substituents of the rosarrin enabled to isolate a 25*π*-electron species as air stable form [10]. Furthermore, upon varying the *meso*-substituents from pentafluorophenyl group to bis(trifluoromethyl)phenyl group displayed strong, intermolecular ‘face-to-face’, π-π interaction between antiaromatic molecules. Formation of the ‘face-to-face’ stacked dimer showed temperature dependency with intriguing photophysical properties [11]. However, experimental exploration of the rosarrins often encountered difficulties because varying *meso*-substituents are synthetically challenging due to low yield and difficult purification process. Thus, a viable strategy would be the post-synthetic modification of the parent rosarrin. This strategy was successfully implemented in some porphyrin systems also. Aromatic nucleophilic substitution (S_N_Ar) of the *meso*-(pentafluorophenyl) group with diverse ranges of nucleophiles such as alcohols, amines, and thiols has been performed to afford modified porphyrins [12,13,14,15,16,17,18]. We envisaged to exploit this strategy to the modification of rosarrin to develop new rosarrins and to explore their photophysical and redox chemistry. Towards this, herein we report a synthesis of *meso*-tris-(1,2,4-trifluorodibenzo(*b,e*)(1,4)dioxine) substituted rosarrin (**3**) by a regio-selective aromatic nucleophilic substitution reaction of rosarrin (**2**).

## 2. Results and Discussion

**Scheme 1:**
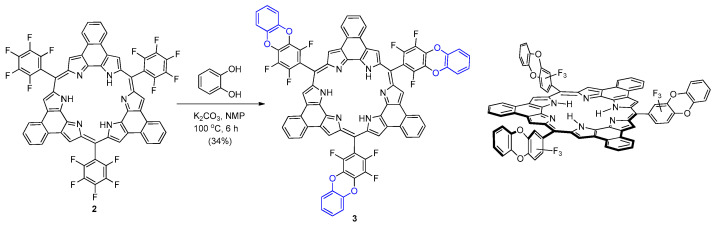
Synthesis of rosarrin **3** (NMP: *N*-methylpyrrolidone).

The synthesis of starting rosarrin **2** was performed by acid-catalyzed condensation of naphthobipyrrole with pentafluorobenzaldehyde utilizing the reported procedure [4]. Rosarrin **2** was then treated with catechol in the presence of base (K_2_CO_3_) in *N*-methylpyrrolidone (Scheme 1). As shown in Scheme 1, the reaction unexpectedly resulted in the exclusive formation of double nucleophilic aromatic substitution product **3** [12,13]. One phenolic –OH group in the catechol reacted region-selectively with the 4-fluoro group first. Then the remaining phenolic hydroxyl group reacted with adjacent fluoro-group by intramolecular nucleophilic aromatic substitution. However, our attempts with phenol and benzyl alcohol to yield their corresponding derivatives were futile.

**Figure 2:**
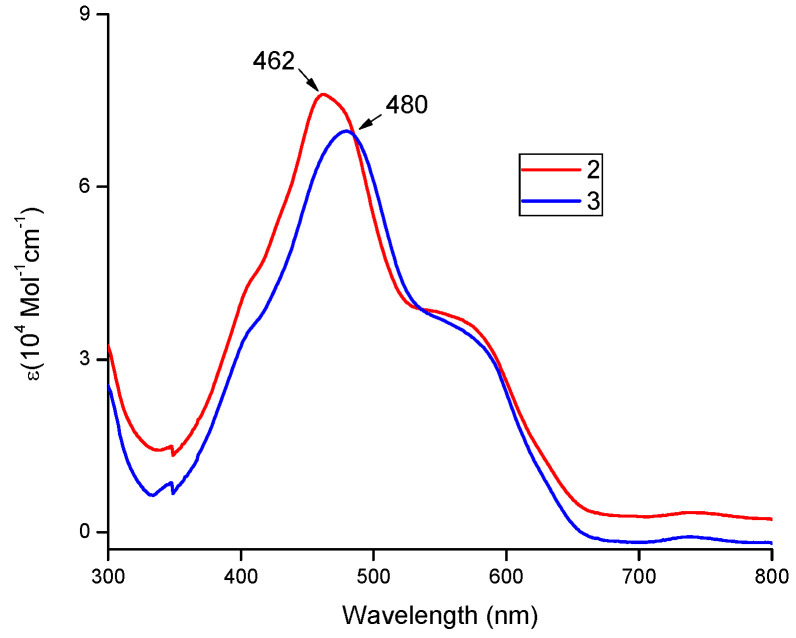
UV-Vis spectra of rosarrin **3** (blue line) and rosarrin **2** (red line) in CH_2_Cl_2_.

The rosarrin **3** is isolated by repeated column chromatography resulting in 34% yield of pure product, which was characterized by standard spectroscopic techniques. ^1^H-NMR spectrum of **3** (in CDCl_3_) showed the indolic C-H protons at 4.7 ppm whereas the phenylene protons appeared at 6.9 ppm as multiplets (Supplementary Material). Notably, the signal of the core N-Hs appeared at ~26.0 ppm indicating the presence of a strong paratropic ring current. The UV-Vis absorption spectra of **3** exhibited a broad absorption band at 480 nm (ε = 6.9 × 10^4^ M^−1^cm^−1^) which is in similar with the spectral pattern of rosarrin **2** but the Soret-like band is shifted ~18 nm bathochromically (Figure 2).

Such a larger shift is unique in these classes of macrocycles considering the fact that the *meso*-substituents adopt nearly orthogonal conformation against the rigid macrocyclic core. For instance, peripherally modified rosarrin analogues displayed minimal changes in absorption spectra compared to the parent rosarrin **2** [10,11]. We envisaged that macrocycle core possesses a modified dioxine ring at *meso* position which alters its dihedral angle and results such a bathochromic shift.

**Figure 3:**
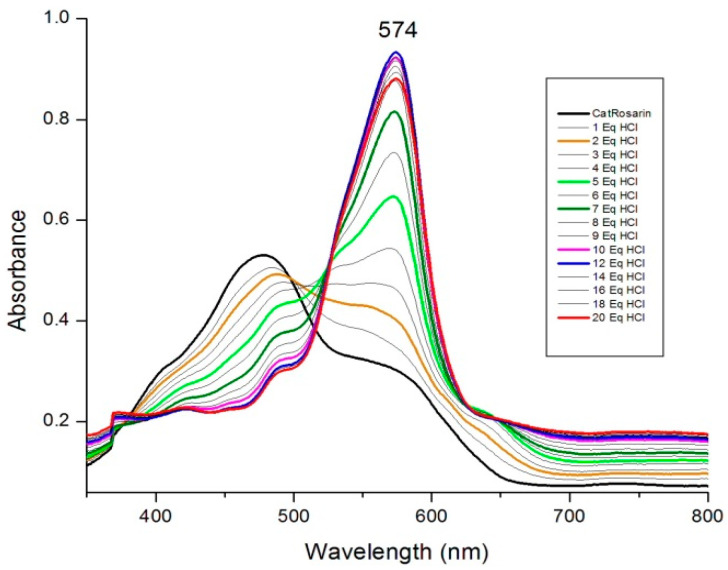
UV-vis absorption spectral change of rosarrin **3** [7.36 × 10^−6^ M] upon addition of HCl in CH_2_Cl_2_.

Protonation and redox behavior of rosarrin **3** were studied by adding various protic acids and spectral changes were monitored through absorption spectroscopy. Rosarrin **3** displayed proton coupled electron transfer behavior just as observed in case of rosarrin **2** [8]. Addition of 10 equivalents of trifluoroacetic acid (TFA) to the solution of **3** (in dichloromethane) showed no appreciable spectral changes. However, significant bathochromic shift of the absorption maxima from 480 nm to 515 nm was observed upon addition of excess TFA (~1000 equiv.). This spectral changes suggested the formation of tri-protonated species of (**3•H_3_^3+^**) as previously noted in the case of rosarrin **2**. Such protonation equilibrium reaction is expected in case of redox-resistant trifluoroacetic acid [19]. However, titration of rosarrin **3** with HCl in CH_2_Cl_2_ showed dramatic changes in absorption maxima. As shown in Figure 3, gradual appearance of an intense band at 574 nm and disappearance of 480 nm band was noted simultaneously upon addition of 10 equiv. HCl. This spectral change ascribed to the formation of one electron reduced 25π dication radical species of rosarrin **3** [8]. However, we could not identify the actual reductant species for this one electron reduction process. On the other hand, two-electron reduction of rosarrin **3** to the corresponding 26π electron system is achieved with addition of slight excess HI (~5 equiv.). Simultaneous appearance of the characteristic Soret-like band at 613 nm while disappearance of the 480 nm band are typical indication of two-electron reduction of rosarrin **2** [8]. These observations indicate that the stepwise nature of proton couple electron transfer in rosarrin **3** is well stands in line with the pattern noted in case of **2**.

Surprisingly, we observed that upon titration with perchloric acid (HClO_4_, up to ~5 equiv.), new absorption band at 574 nm gradually appears along with new band at 620 nm with isosbestic point at 500 nm (SI). These observations indicate that in presence of perchloric acid, 24π-, 25π- and 26π-electron species co-exist in equilibrium under this experimental condition. The presence of 25π-odd electron species was confirmed by taking EPR spectroscopy of the solution of **3** in the presence of perchloric acid. The solution clearly showed the signal without fine splitting supporting the observations. Formation of the radical species was also confirmed in ^1^H-NMR spectra. When the solution of **3** mixed with excess perchloric acid in CDCl_3_, complete disappearance of all signals was observed (SI).

**Scheme 2:**
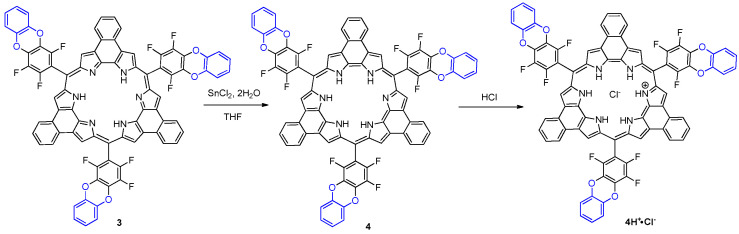
Synthesis of aromatic rosarrin **4** and **4H^+^•Cl**^−^ from **3** (THF: tetrahydrofuran).

**Figure 4:**
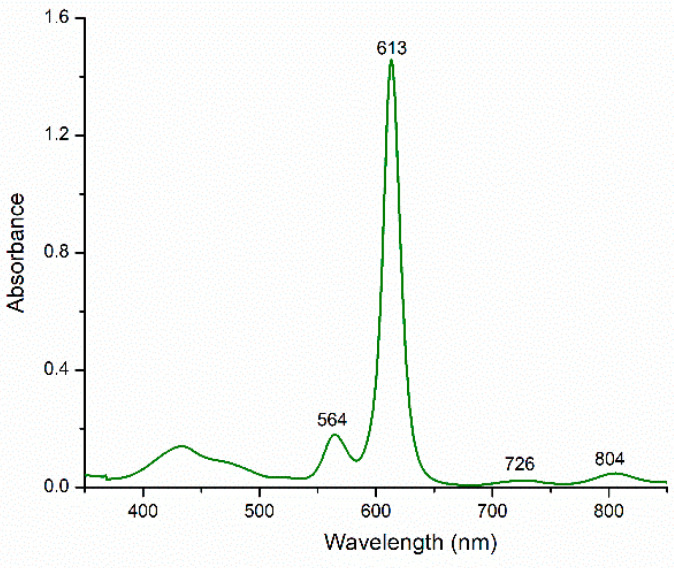
UV-Vis absorption spectra of **4H^+^•Cl**^−^ in CH_2_Cl_2_ (conc. = 1.8 × 10^−6^ M).

The clean two-electron reduction of 24π-electron antiaromatic rosarrin to the corresponding 26π-electron aromatic system has been challenging task due to the unstable nature of the two-electron reduced form. The reduced form easily re-oxidized to corresponding antiaromatic form during work-up period especially when the solution is basic. For example, attempted two-electron reduction of **3** to the corresponding 26π-electron system using an aqueous sodium dithionite cannot afford clean conversion [8]. However, we found that applying SnCl_2_ dihydrate as a reducing agent resulted in efficient conversion to the corresponding 26π electron aromatic form without any contamination of the oxidized form. When rosarrin **3** was treated with SnCl_2_•2H_2_O, clean reduction to 26π electron aromatic rosarrin **4** was achieved, and the reduced product was isolated as HCl salt in pristine pure form under standard column chromatography (Scheme 2). The identity of the rosarrin **4** could be easily confirmed by ^1^H-NMR spectra. Typically, the core-N-Hs appeared at −5.33 ppm as broad singlet which is in comparison with those observed for rosarrin **2**. The significant downfield shift of the indolic C-Hs (Δδ = 6.2 ppm) in comparison with those in **3** clearly confirm the aromatic nature of rosarrin **4**. Absorption spectra of **4H^+^•Cl**^−^ exhibited a strong Soret band at 613 nm and Q bands with clear vibronic structures as shown in Figure 4. We proposed that electronic effect of the fused benzodioxane rings contribute to the stabilization of the 26-π system.

**Figure 5:**
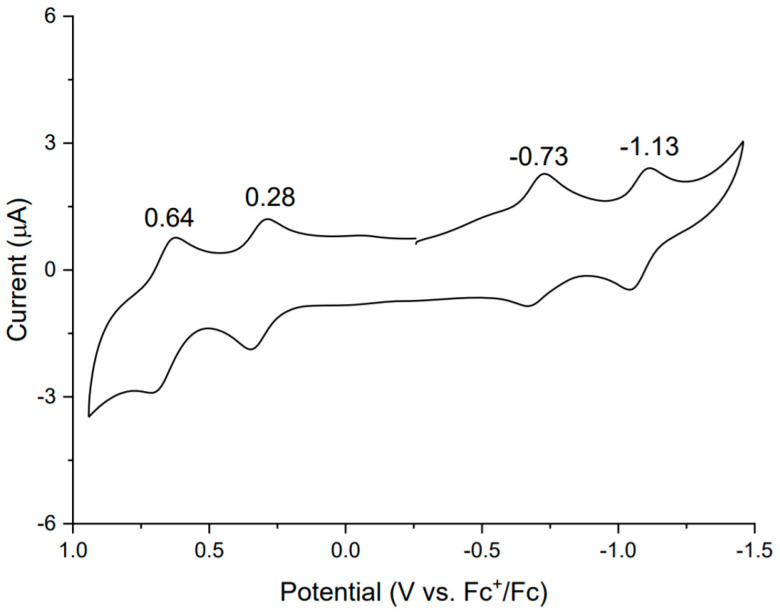
Cyclic voltammogram of **3** in TBAPF_6_ [0.1 M] in CH_2_Cl_2_ with scanning rate of 100 mV/s.

Figure 5 shows a cyclic voltammogram of rosarrin **3** in dichloromethane solution. Rosarrin **3** displayed a typical rosarrin voltammogram with four reversible redox waves with a peak at −0.73, −1.13, 0.64 and 0.28 volts. Observed potentials are quite similar to rosarrin **2 [8]** which confirms that the post synthetic modulation such as **3** do not change the inherent redox properties and displays same electronic reversibility.

## 3. Materials and Methods

All commercially available reagents and compounds were used as received without further purification. Naphthobipyrrole moiety was synthesized according to a literature procedure [20]. All solvents were dried and distilled before going to use. All the reactions, which are sensitive to moisture or oxygen were performed under a nitrogen atmosphere. The thin layer chromatography was performed on aluminum backed silica gel 60 F254 (Merck) or neutral alumina F254 (Sigma-Aldrich) (Darmstadt, Germany). Product purification was performed by column chromatography on silica (230–400 mesh, MN) or neutral aluminum oxide 90 (70–230 mesh, Merck). Synthesized compounds identity and purity was confirmed by using 400/ 600 NMR spectroscopy (Carnation, WA, USA). Jeol 400MHz was used to check ^1^H-NMR, variable NMR study and TFA titrations. For ^13^C-NMR spectroscopy Bruker 600 MHz was used. Mass spectral data were obtained by Voyager DE-STR MALDI-TOF (Foster City, CA, USA). Absorption spectra were recorded with a Varian CARY 100 Conc spectrometer (Santa Clara, CA, USA). Cyclic voltammograms were performed on CHI660E Electrochemical Workstation (CH Instruments, Inc., Austin, TX, USA) Three-electrode cells were comprised of a platinum disk electrode as the working electrode, a platinum wire as the counter electrode and a platinum wire as reference electrode. The reference potential was calibrated with ferrocene/ferrocenium (Fc/Fc^+^). The electrolyte solution was dichloromethane (DCM) containing 0.1 M tetrabutylammonium hexafluorophosphate (TBAPF_6_), which was bubbled with nitrogen gas prior to electrochemical tests.

### 3.1. Synthesis of Rosarrin **3**

Rosarrin **2** (50.0 mg, 45 μmol), K_2_CO_3_ (48.0 mg, 347 μmol) and catechol (45.0 mg, 405 μmol) were charged in an oven dried, nitrogen cooled round bottom flask and evacuated for one hour. After flushing with nitrogen, *N*-methylpyrrolidone (~6.0 mL) is added and reaction mixture flushed with vacuum and nitrogen three times. Reaction mixture was shielded from light by aluminum foil and heated at 100 °C for 6 h. Reaction mixture was cooled and transferred to the separating funnel, water was added and extracted with ethyl acetate. After drying over anhydrous sodium sulfate, organic layer removed under low pressure and residual solid is passed through silica gel. The pure product was obtained by column chromatography on silica (DCM/Hexane = 1/1) in 34% yield. ^1^H-NMR: (400 MHz, CDCl_3_) δ (ppm) δ 7.0–6.88 (m, 24 H), 4.73 (s, 6H, β-H); ^13^C-NMR (125 MHz, CDCl_3_) (ppm) 151.0, 140.3, 139.9, 131.6, 129.0, 126.4, 125.5, 125.2, 123.5, 123.4, 117.1, 116.9; ^19^F-NMR (376 MHz, CDCl_3_) δ (ppm) −139.47 (dd, *J* = 7.5, 22.5 Hz), −141.11 (d, *J* = 18.8 Hz), −160.82 (d, *J* = 22.5 Hz); MALDI-TOF MS Calculated for C_81_H_33_F_9_N_6_O_6_ *m/z* = 1356.23; Found 1357.389 (M + 1). UV-Vis (in CH_2_Cl_2_) λ [nm] (ε [M^−1^cm^−1^]) 480 (6.9 × 10^4^).

### 3.2. Reduction of Rosarrin **3** to **4H^+^•Cl^−^**

Rosarrin **3** was dissolved in dry THF and degassed with argon for ten minutes. SnCl_2_·2H_2_O (5 equiv.) was added to it. The reaction mixture was allowed to stir at room temperature under protection from light. Solution was gradually turned deep green color indicating formation of corresponding 26-pi species. Upon completion of the reaction (2h), solvent was evaporated in rotary and crude was subjected to silica gel column chromatography using DCM/Hexane (3/2) as eluent. The product **4H^+^•Cl**^−^ was obtained as dark red solid in 65% yield. ^1^H-NMR: (400 MHz, CDCl_3_) δ 10.97 (s, 6H, β-H), 9.81 (brs, 6H, Ar-H), 8.23 (brs, 6H, Ar-H), 7.40–7.38 (m, 3H, Ar-H), 7.19–7.26 (m, 9H, Ar-H) (ppm); ^13^C-NMR (125 MHz, CDCl_3_) (ppm) 151.6, 141.2, 140.8, 135.8, 131.4, 131.1, 130.2, 128.5, 127.1, 125.7, 125.6, 125.4, 188.7, 117.5, 117.4, 115.5, 96.0; MALDI-TOF MS Calculated for [C_81_H_36_F_9_N_6_O_6_]^+^ *m*/*z* = 1359.25; Found 1359.359 (M^+^), UV-Vis (in CH_2_Cl_2_) λ [nm] (ε [M^−1^cm^−1^]) 613 (8.1 × 10^5^).

## 4. Conclusions

Post synthetic modification of *meso*-substituents of antiaromatic hexaphyrin (1.0.1.0.1.0) **2** has achieved for the first time by utilizing double nucleophilic aromatic substation reaction. The resulting rosarrin **3** displayed significant bathochromic shift in absorption maximum compared with the parent compound **2**. Acid titration of **3** clearly shows that **3** can be partially reduced or fully reduced to aromatic system by proton coupled electron transfer mechanism. Clean reduction from rosarrin **3** to **4H^+^•Cl**^−^ could be achieved adopting SnCl_2_ as reducing agent. The reduced rosarrin **4H^+^•Cl**^−^ displayed full aromatic characters corresponding to the 26π electron system. Electrochemical results clearly shows the retention of redox behavior after post synthetic modification at *meso*-position. Currently, efforts are being devoted towards the stabilization of 22π-electron system.

## Data Availability

The data presented in this study are available on request from the corresponding author.

## Supplementary Materials

The following are available online. Figure S1: ^1^H-NMR of **3**, Figure S2: MALDI-TOF spectrum of **3**, **Figure S3**: ^13^C-NMR of **3**, Figure S4: ^19^F NMR of **3**, **Figure S5**: Low temperature ^1^H-NMR of **3**, Figures S6,S7: UV-vis absorption spectral of **3** with TFA, Figure S8: UV-vis absorption spectral of **3** with TFA, Figure S9: UV-vis absorption spectral of **3** with HI, Figure S10: Conversion rate of **2** and **3** to respective 26π analogues, Figure S11: UV-vis absorption spectral of **3** with HClO_4_, Figure S12: ^1^H-NMR spectra of **3** with HClO_4_, Figure S13: EPR spectrum of **3** with HClO_4_, Figure S14: ^1^H-NMR spectra of **4H^+^****•Cl**^−^, Figure S15: MALDI-TOF spectrum of **4H^+^****•Cl**^−^, Figure S16: ^13^C-NMR of **4H^+^****•Cl**^−^, Figure S17: UV-vis absorption spectra of **4H^+^****•Cl**^−^.

## Sample Availability

Samples of the compounds **3** and **4H^+^****•Cl**^−^are available from the authors.
