# Supplementary material for: Post Synthetic Modification of Planar Antiaromatic Hexaphyrin (1.0.1.0.1.0) by Regio-Selective, Sequential SNAr
Source: Molecules. 2021 Feb 15;26(4):1025. doi: 10.3390/molecules26041025 (PMC7919474; doi:10.3390/molecules26041025)
Supplement: Supplementary file 1 [file molecules-26-01025-s001.pdf]

Supplementary Materials

Post Synthetic Modification of Planar Antiaromatic Hexaphyrin(1.0.1.0.1.0) By Regio-selective, Squential S_NAr

*Ranjan Dutta, Brijesh Chandra, Seong-Jin Hong, Yeonju Park, Young Mee Jung and Chang-Hee Lee**

Department of Chemistry, Kangwon National University, Chun Cheon, 24341Korea

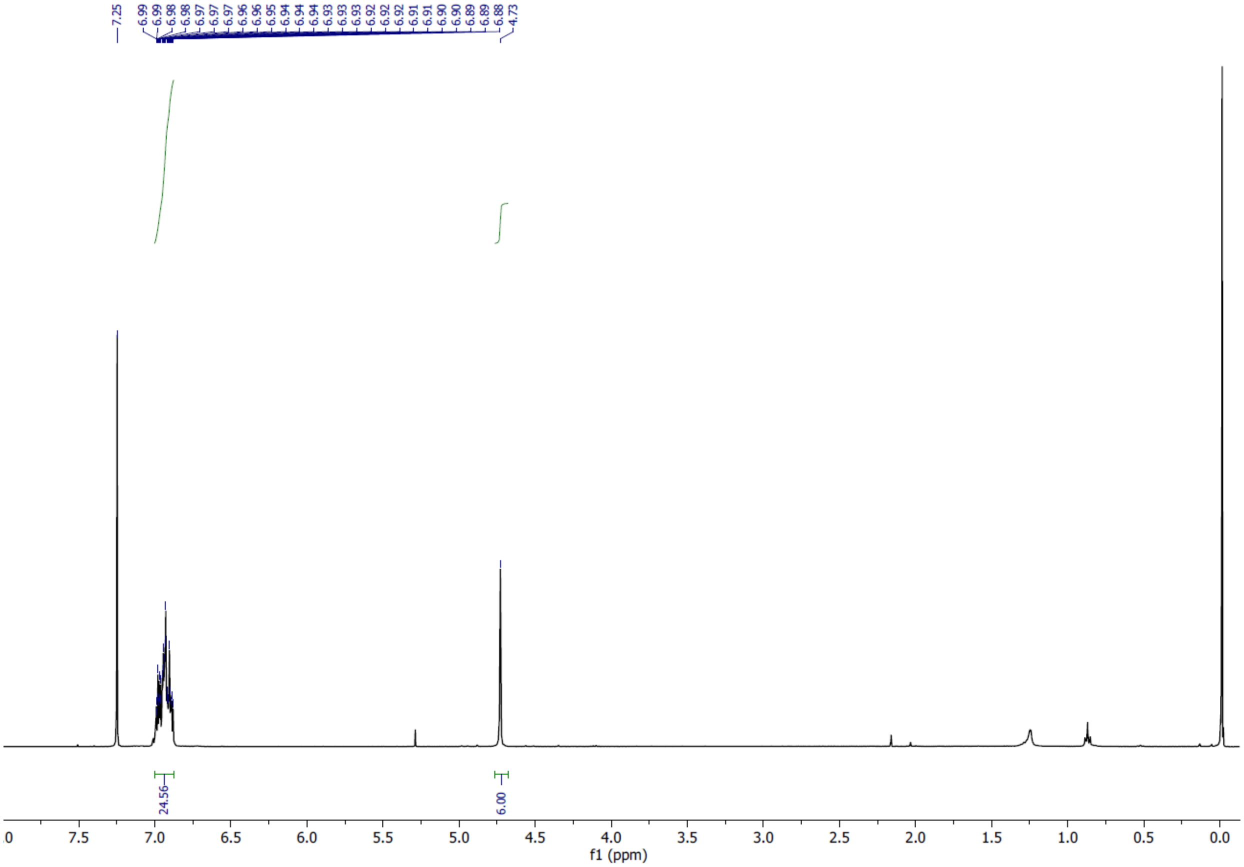

Figure S1. ^{1}H NMR of **3** in $CDCl_3$.

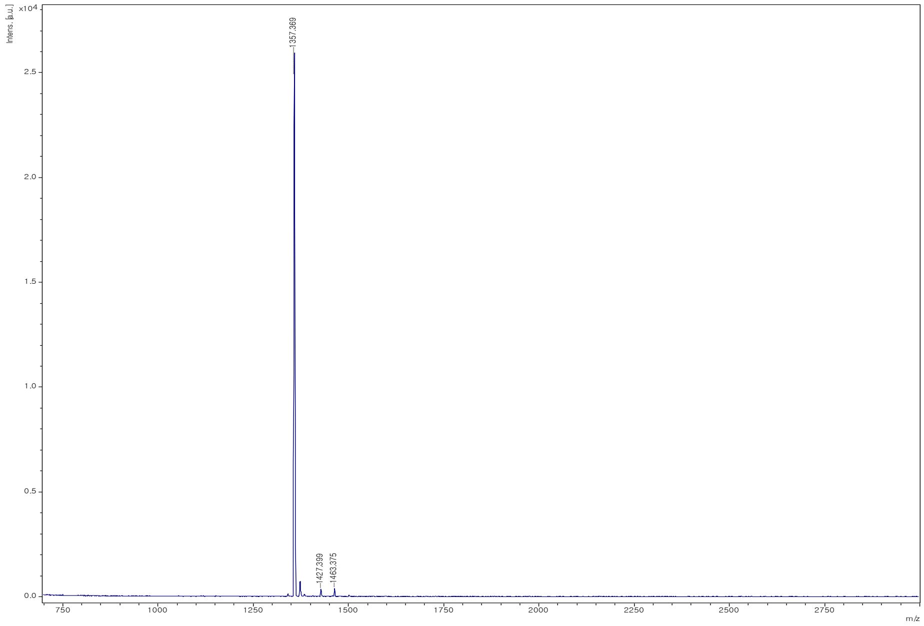

Figure S2. MALDI-TOF spectrum of **3**.

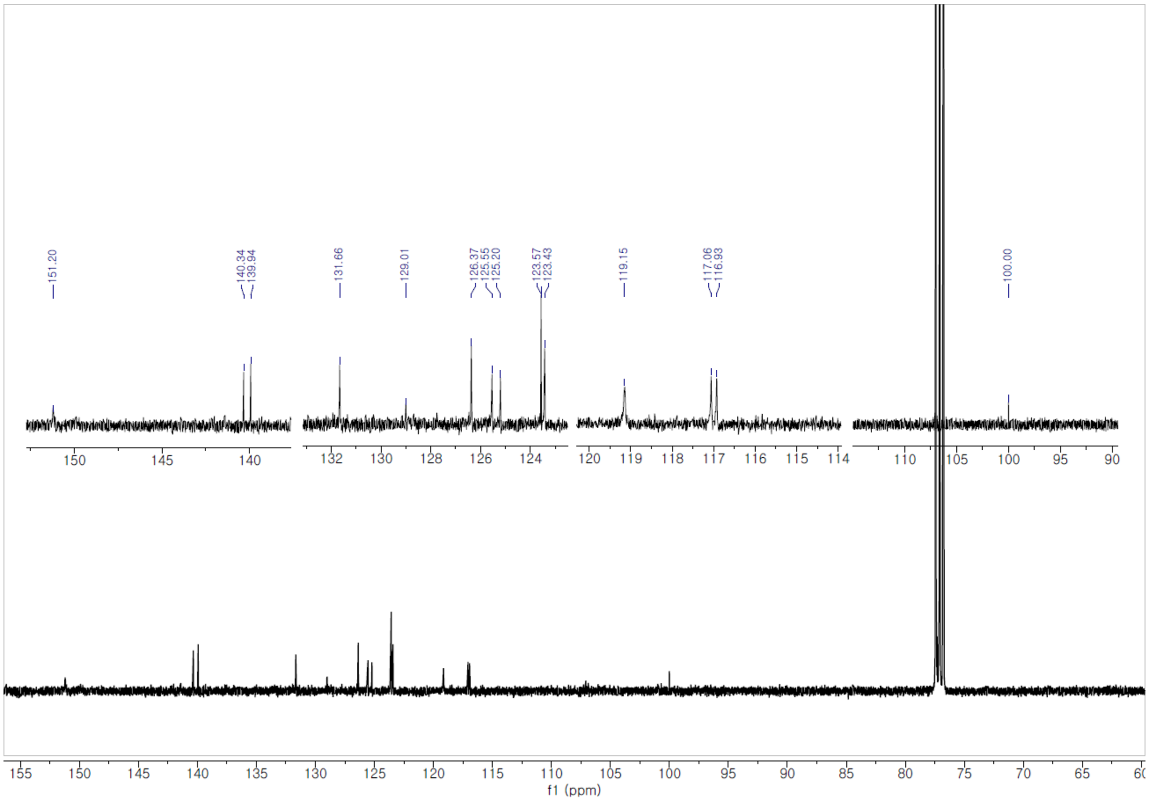

Figure S3. ^{13}C NMR of **3** in $CDCl_3$ (expanded region is included).

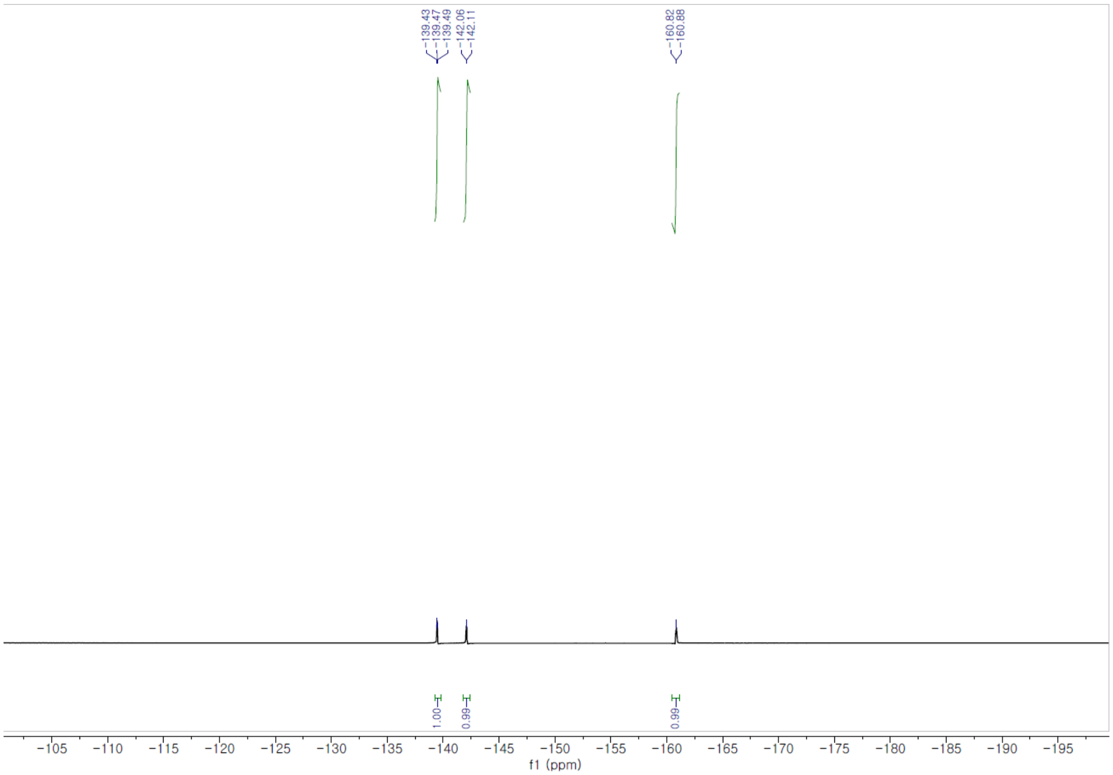

Figure S4. ^{19}F NMR of **3** in $CDCl_3$.

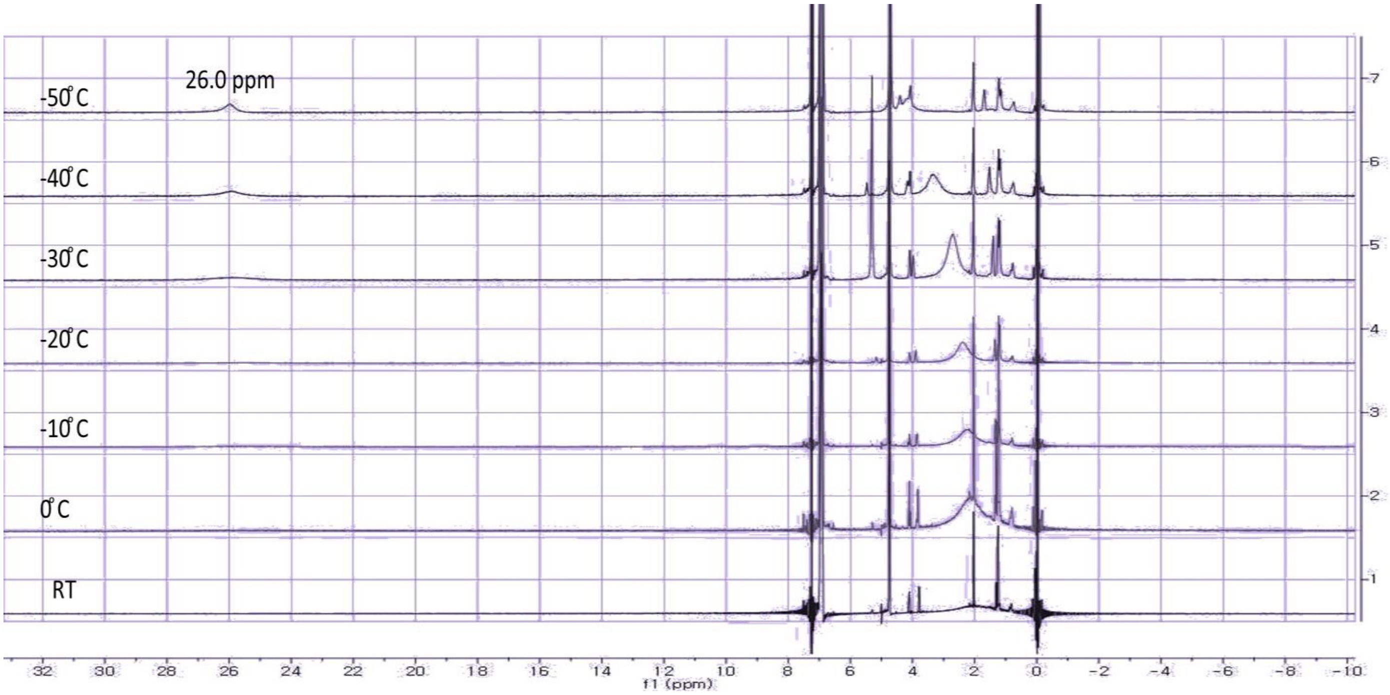

Figure S5. Low temperature ^{1}H NMR of **3** in $CDCl_3$.

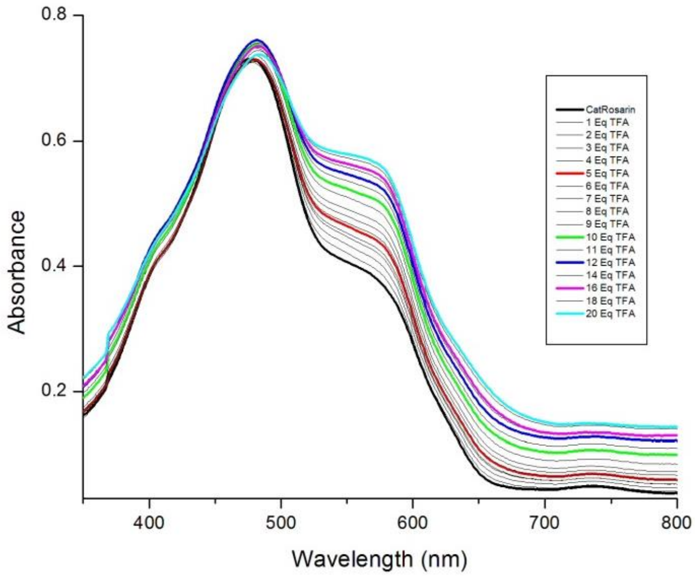

Figure S6. UV-vis absorption spectra of **3** [7.36 x 10^{-6} M] upon addition of TFA (20 eqv.) in CH_2Cl_2.

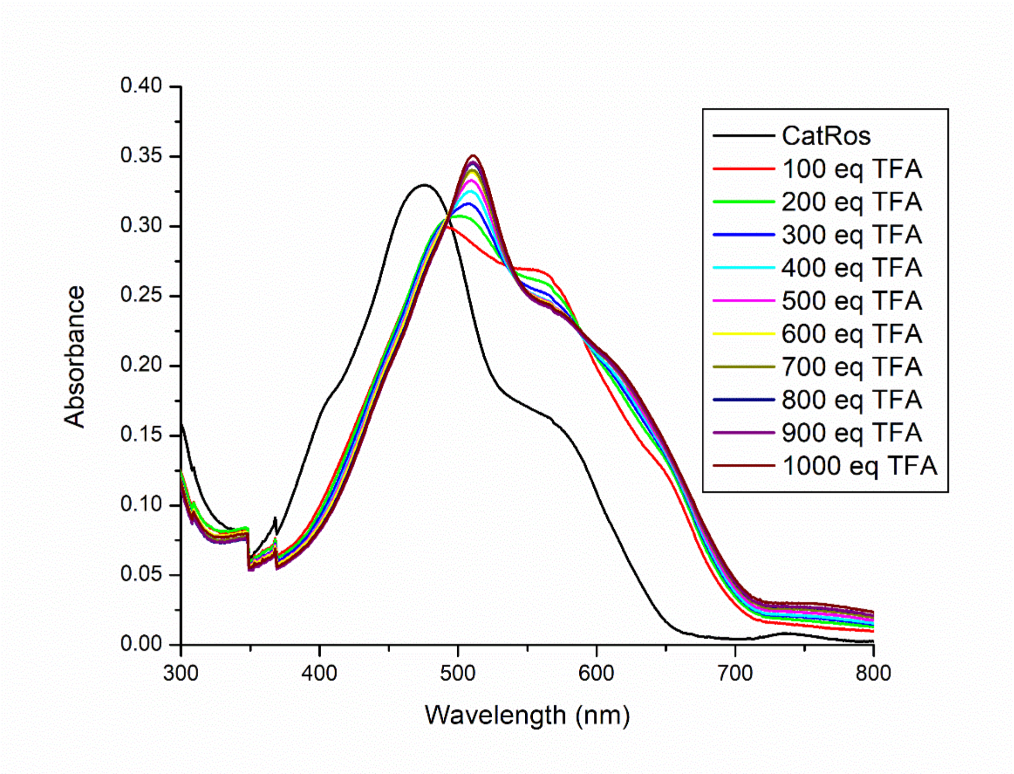

Figure S7. UV-vis absorption spectra of **3** [7.36 x 10^{-6} M] upon addition of TFA (1000 eqv.) in CH_2Cl_2.

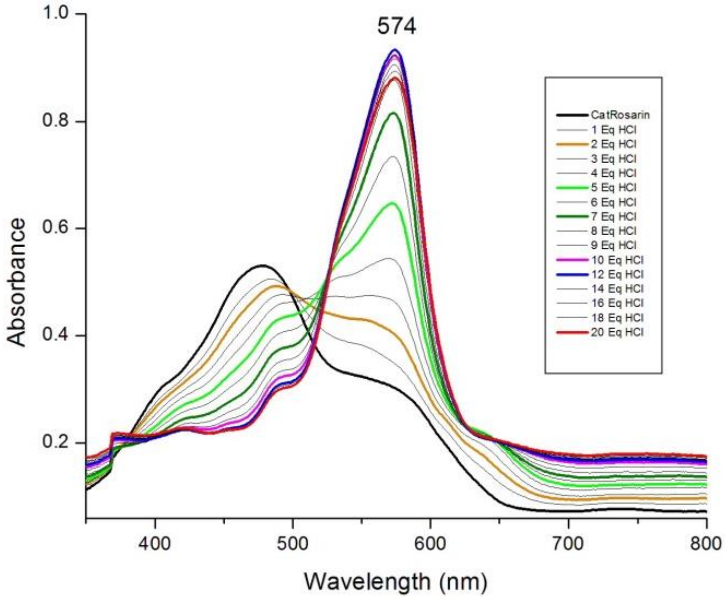

Figure S8. UV-vis absorption spectra of **3** [7.36 x 10^{-6} M] upon addition of HCl in CH_2Cl_2.

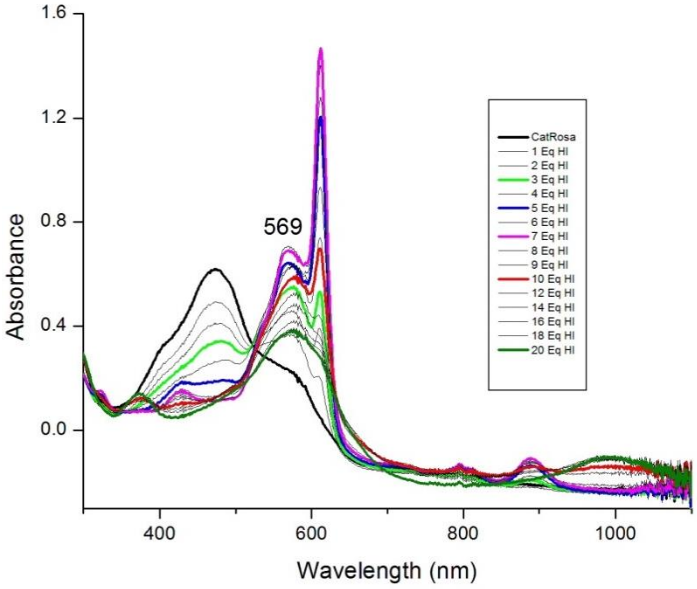

Figure S9. Change in UV-vis absorption spectra of **3** [7.36 x 10^{-6} M] upon addition of HI in CH_2Cl_2.

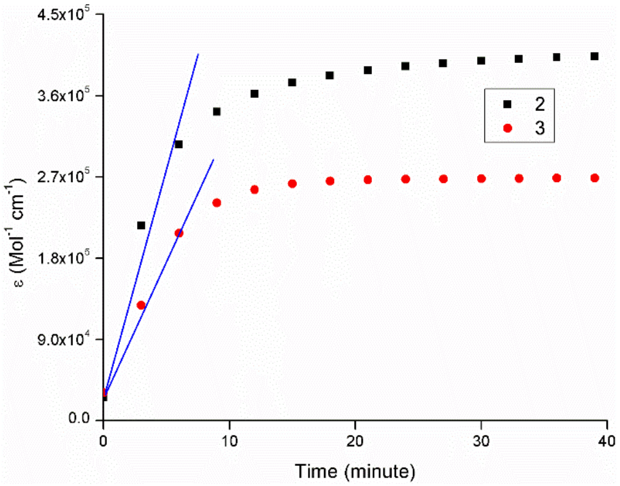

Figure S10. Conversion rate of **2** and **3** to their respective 26-pi analogues with 3eqv. HI in CH_2Cl_2 followed at 613 nm.

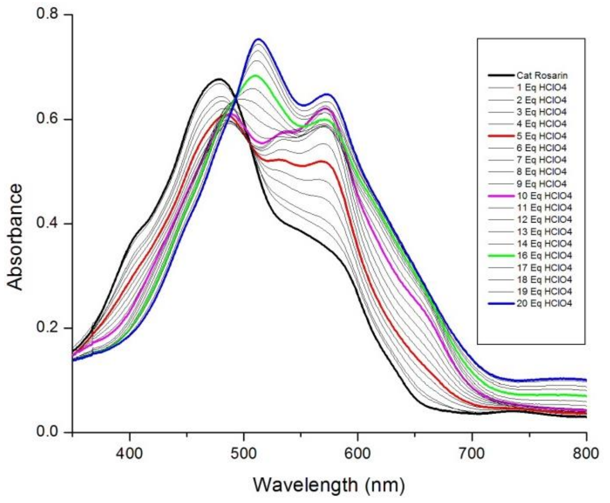

Figure S11. UV-vis absorption spectrum of **3** [7.36 x 10^{-6} M] upon addition of $HClO_4$ in CH_2Cl_2.

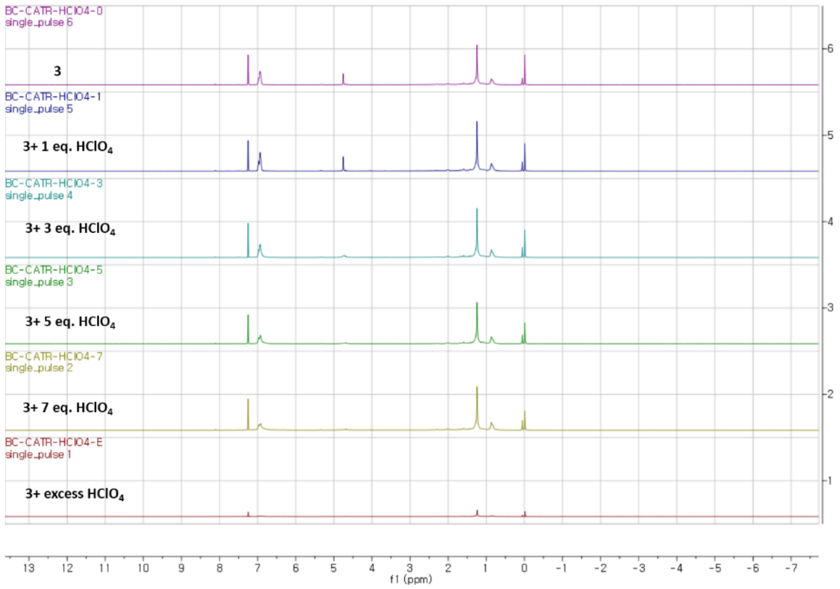

Figure S12. ^{1}H NMR spectra of **3** upon addition of $HClO_4$ in $CDCl_3$.

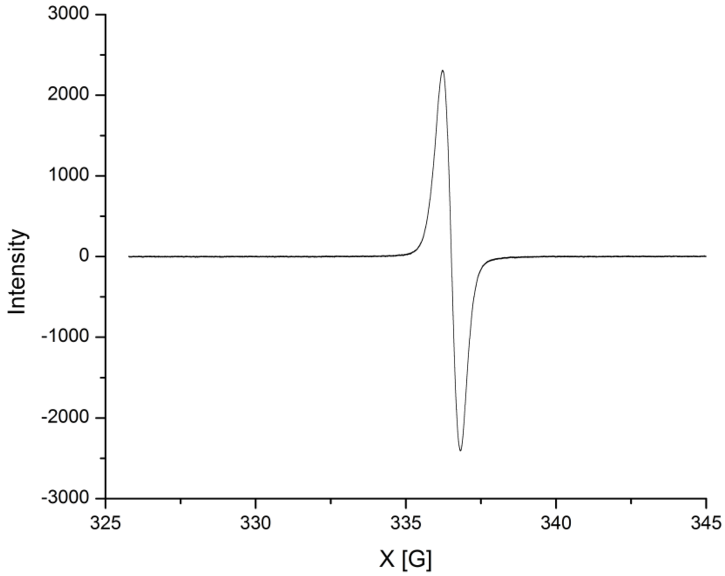

Figure S13. EPR spectrum of **3** upon addition of excess $HClO_4$ in CH_2Cl_2.

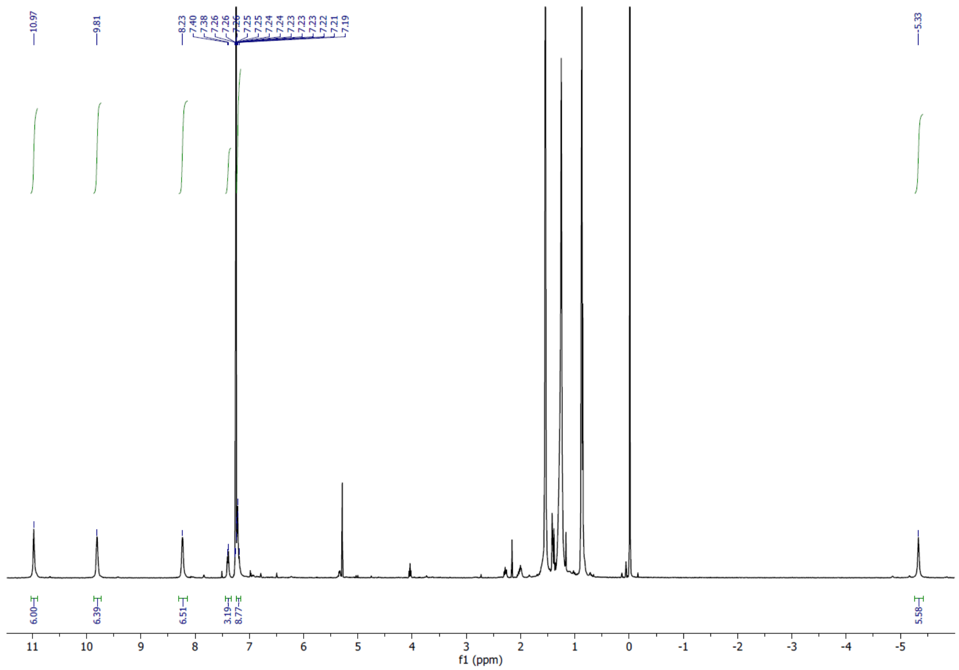

Figure S14. ^{1}H NMR spectra of **4H^{+}•Cl^{-}** in $CDCl_3$.

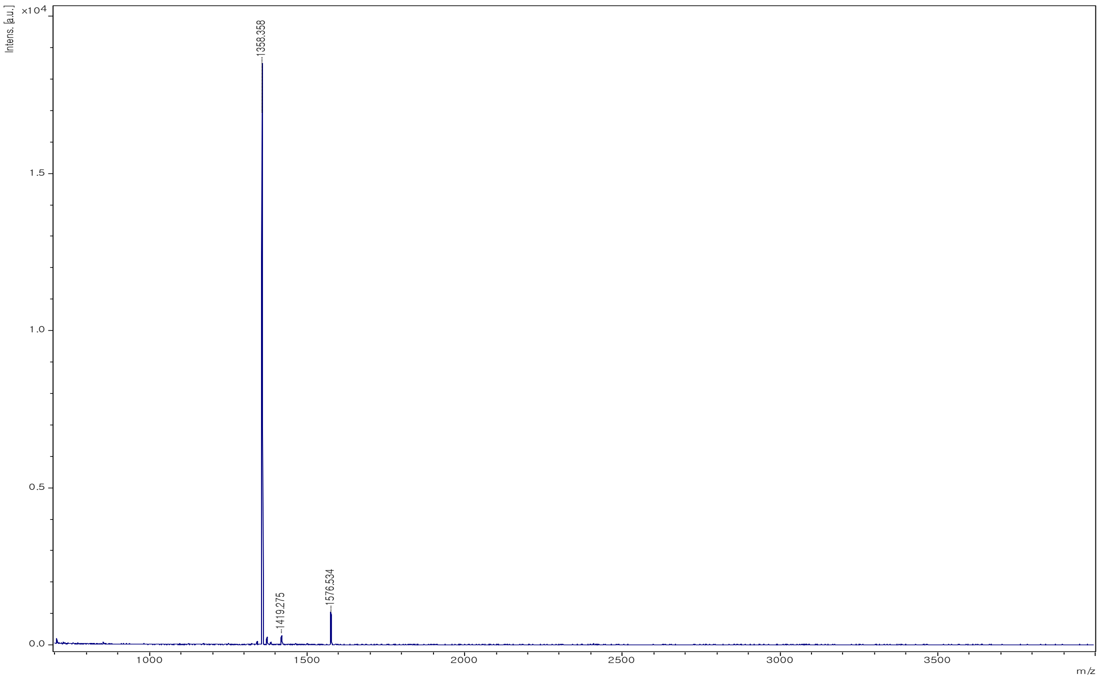

Figure S15. MALDI-TOF spectrum of **4H^{+}•Cl^{-}**.

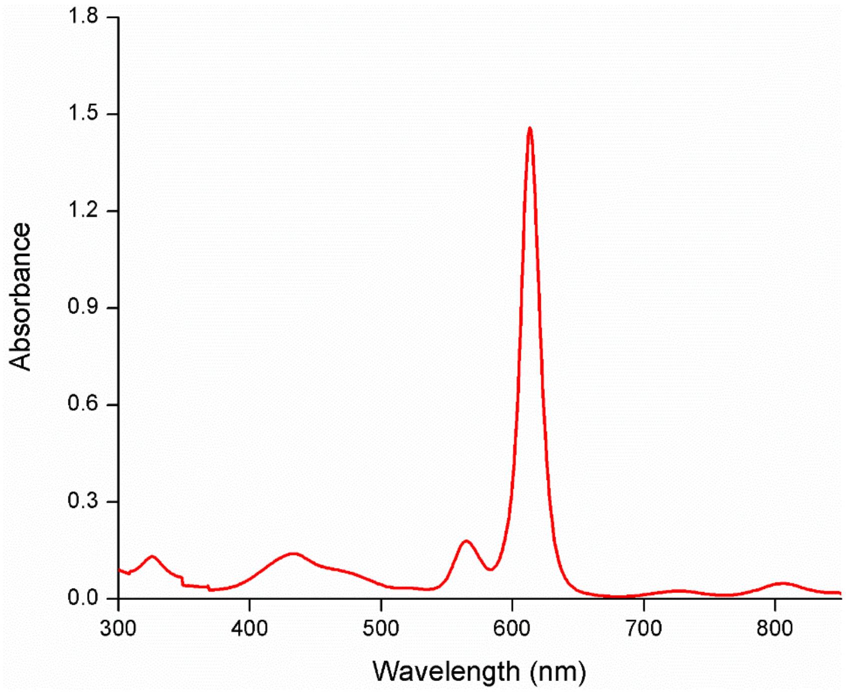

Figure S16. UV-vis absorption spectra of **$4H^{+}$•Cl^{-}** [1.8 x 10^{-6} M] in CH_2Cl_2.
